# Economic stress of people 50 + in European countries in the Covid-19 pandemic–do country policies matter?

**DOI:** 10.1007/s10433-021-00662-2

**Published:** 2021-11-24

**Authors:** Agnieszka Chłoń-Domińczak, Dorota Holzer-Żelażewska

**Affiliations:** SGH Warsaw School of Economics, Warsaw, Poland

**Keywords:** Covid-19 pandemic, Economic risks, Older population, SHARE

## Abstract

The Covid-19 pandemic caused lockdown of economies, which in turn led to the worsening of the economic situation of many households. During the first wave of the Covid-19 pandemic governments undertook various measures to support economies and societies, including jobs protection along with financial support provision to people who suffered financial loss during the economic crisis. We analyse the economic situation of older Europeans, depending on their socio-economic status as well as country of residence characteristics, including economic and labour market changes during the first phase of the pandemic, the strictness of government policies but also the country development level using the Human Development Index. We use the results of the Survey of Health, Ageing, and Retirement in Europe (SHARE), including the SHARE Corona Telephone Survey, which was conducted during the first wave of the Covid-19 pandemic. Our results indicate that individual characteristics have a higher impact on individual economic stress, compared to country characteristics. However, country’s response to the consequences of the Covid-19 pandemic, but also the overall level of development influences the economic situation and ability to cope with the economic risks people aged 50 and over face. People in more developed countries have smaller difficulties in making ends meet, while the economic crisis and more stringent policies reduce chances to receive financial support and increase economic risks.

## Introduction

In response to the Covid-19 pandemic outbreak, governments introduced lockdown measures that affected the labour market and economic situation of many Europeans. Many industries were forced to reduce employment due to the closures. Many households, especially those who had already faced difficulties with making ends meet, experienced a further increase of economic stress due to the loss of employment income, and an increased dependence on financial support provided by employers, from public sources or private transfers.

The European countries were affected differently by the Covid-19 pandemic; they also introduced different policies that influenced their economic growth and labour market. These policies and their outcomes, in turn, affected the economic situation of European populations. Our main contribution is to provide evidence on the role of government intermediate policies, changes in the economic situation in the country as well as the overall country development on the economic stress of people aged 50 years or over in Europe. This gives a further insight on if and how people in the second half of their life course are protected against economic stress in the crisis situation caused by the Covid-19 pandemic.

Our results are based on the responses from SHARE Corona Telephone Survey that was conducted during the first wave of pandemic, from the beginning of June 2020. The use of harmonised SHARE Corona Telephone Survey data as well as ability to use longitudinal information from earlier SHARE Wave 7, gives a unique opportunity to measure and interpret the observed differences in a cross-country dimension, including 25 European countries. The obtained data reveal that there are large differences between the countries with regard to different aspects of economic situation of older people. These differences concern the entire analysed population, also people who were working before the Covid-19 outbreak and faced the risk of job loss. Finally, it is also important to see, how people who are most vulnerable economically, particularly those who had problems before the pandemic, adapt to their worsening situation in order to meet their current consumption needs.

In our analysis, we focus on the economic situation of the above-mentioned groups in the European countries and how it relates to individual and country characteristics. Our contribution to the literature is that we provide cross-country comparisons based on internationally harmonised data, but also that we analyse different symptoms of economic stress of the older Europeans during the first wave of the Covid-19 pandemic, which is not jointly discussed in the literature.

Our hypothesis is that the overall country’s development prior to the crisis, short-term economic outcomes, the severity of the Covid-19 pandemic and undertaken direct policy measures, have a significant impact on the symptoms of economic stress, including the risk of job loss, economic situation of households of people in age group 50 or over, including the most vulnerable as well as their access to financial support.

The article is structured as follows. First, we present the findings on the impact of crisis on the situation of people 50 + , including the Great Recession and first evidence from the Covid-19 crisis. We also present main statistical indicators showing differences in the economic and labour market outcomes in 2020 that we use as the independent variables in the analysis. Then, we present our analytical approach and findings related to the economic situation of the analysed population during the first wave of the pandemic. We discuss the role of individual and country characteristics in explaining the analysed aspects of the economic situation of people aged 50 or over. Finally, we present the discussion of the results and conclusions, including policy implications.

## Economic situation of older people–what can we learn from the previous crises and what is the potential impact of the current Covid crisis?

While the evidence on the impact of the Covid-19 pandemic on the economic situation of the population aged 50 and over is still limited, there is a growing body of literature that indicates risks that are emerging for this group. Miller 2020 underlines that the Covid-19 pandemic has affected older adults and their families, caregivers, and communities, through the economic risks that were faced by workers, retirees, and the inactive population. A lot of attention is focussed on the economic situation and labour market status of older workers. There are also some important lessons that can be learnt from previous crises, including the Great Recession, which are valuable to understand the current economic risks for older workers and pensioners.

With respect to the labour market situation, Johnson (2012) provides evidence that the negative labour demand shocks increase employment discrimination against both, current and prospective older employees. The lessons from previous recessions, particularly from the recent Great Recession, also show that older workers have significant difficulties in returning back to employment, which means that they also face a higher risk of being long-term unemployed or shifting to inactivity (Bui et al. 2020; Johnson and Butrica 2012). The evidence from the US labour market indicates that older workers, in particular women, were more affected by the Covid-19 recession, compared to the previous crises.

There are several reasons why the impact of the Covid-19 crisis on the employment of older workers can be more acute. As pointed out by Ghilarducci and Farmand (2020), older workers more frequently work in essential occupations (i.e. food distribution, home and personal care, cleaning services, transport), and therefore, they are more exposed to the health risk during a pandemic. Evidence from the English Longitudinal Study of Ageing (ELSA) Covid-19 study, conducted in June–July 2020, indicates that people aged 65 and older are the second largest proportion of employees in the shutdown sectors (after the youngest group) (Crawford and Karjalainen 2020). Moreover, older workers frequently work in jobs where teleworking is not an option (Kanfer et al. 2020; Li and Mutchler 2020). A recession can also accelerate routine-biased technological change, as it happened during the Great Recession. This affects older workers more, as they have a lower ability to adjust to these changes, which are intense during recessions (Hershbein and Kahn 2018).

Older workers also face higher risk of job loss due to employers’ preferences. Evidence from previous research shows that employment reductions during a crisis affect workers who have early retirement packages, which increases intergenerational tensions (van Dalen and Henkens 2013). Older workers are also labelled as vulnerable and being at a higher risk in terms of Covid-19, which also increases the propensity of employers to reduce employment in this group (Ayalon et al. 2020). Evidence shows that discrimination against older workers increased during previous recessions (Johnson and Butrica 2012). Results of ELSA Covid-19 study show that around a quarter of older workers were worried about their job security (Crawford and Karjalainen 2020).

The perception of economic and other risks, particularly during the first wave of the pandemic, were also dependant on the socioeconomic situation of individuals. This includes, among others, living in geographical areas with a higher infection rate, which may co-occur with high economic insecurity (Li and Mutchler 2020) or health status which forces job resignation, particularly in sectors that require direct contacts with other people and cannot be performed as telework. Evidence from the ELSA Covid-19 study shows that one in six individuals in England had concerns about their health.

Therefore, the Covid-19 pandemic may trigger a process of self-regulation related to rapidly changing working conditions. Many older workers may engage in activities that enhance their technological skills in order to adjust to the new requirements in their jobs or, on the contrary, start retirement planning (Kooij 2020; Kooij et al. 2020). Some of the authors, i.e. Akkermans et al. (2020), argue that the Covid-19 pandemic can be considered as a shock that will have both short-term and long-term consequences. In particular, job loss at the end of a working career can have an impact on the physical and mental health (Gallo et al. 2000). Job loss due to adverse labour market conditions can also stimulate a faster transition to retirement (Noor 2012). Additionally, financial markets reaction to the crisis as well as dipping of older people households into their savings to support current income, may lead to an overall reduction of retirement savings. Therefore, the economic consequences of the Covid-19 pandemic may have a long-term impact on the future of households pension income (Bui et al. 2020). Also Hurd and Rohwedder (2010) point out that both the risk of job loss and reduced pension savings can lead to reductions in the future consumption of older people households.

Some of the scholars indicate that the impact of the Covid-19 pandemic depends on the country of residence characteristics. Fana et al. (2020) note that the intensity of economic effects depends on the country specialisation. Countries that rely on low productive service activities and with a low share of public employment tend to be the most hardly hit. This means that the Mediterranean countries may be facing the largest challenges, which is also confirmed by Eurofound (2020).

There are also other risks that emerged during the Covid-19 pandemic, which are particularly relevant to people aged 50 or more. One of the emerging issues is the increased intensity of unpaid care, which is more frequently provided by the people in this age group, particularly in case of older family members. Such developments are reported for example in the UK (Carers UK 2020; Gulland 2020).

There is also some evidence that households changed their spending patterns during the pandemic. After the initial “stockpiling”, spending on restaurants and retail is observed (Baker et al. 2020). Furthermore, the survey by Eurofound (Eurofound 2020) revealed that during the pandemic the share of households having arrears in payments of bills increased, particularly among the unemployed, self-employed, and women. It should be noted that financial arrears are more common among people below age 50 and women are also more vulnerable. There are country differences observed. Highest shares of respondents reporting problems with making ends meet, were noted in Greece and Croatia and lowest share in Denmark. More than half of respondents in the Eurofound survey also stated that they would not be able to maintain their standard of living for longer than 3 months, with no or little savings, particularly in the case of Central and Eastern Europe and Mediterranean countries. The lower share of older people experiencing financial difficulties may stem mainly from their stable pension incomes. The old-age pensioners have their incomes to a large scale protected during a crisis, and the monthly pension is an especially valuable asset for households during the recession. However, (Tabor 2020) underlines that to maintain such role, pension systems need to protect their stability so that retirees can get the pensions that they have accrued.

Summing up, the literature clearly indicates that those people who are economically active are more vulnerable to economic consequences of the crises and the specific nature of the Covid-19 crisis can increase these vulnerabilities. Furthermore, some of the strategies related to coping with the drop in the income from labour, such as using pension savings, can have longer-term consequences. The situation of pensioners is better, as their incomes from benefits remain secure. At the same time, the evidence on the actual unintended consequences of the Covid-19 crisis is limited. Majority of evidence is country-based and the existing international evidence does not cover various aspects of the situation of older people. Given that, the results of the SHARE Corona Telephone Survey provide an opportunity to assess, in an internationally comparable manner, the economic risks that people aged 50 or over faced during the first wave of the pandemic, including their decisions related to dealing with the necessity to finance the current consumption with a reduced household income. Furthermore, we can identify to what extent selected individual and country characteristics have an impact on these risks and decisions, which is not assessed in the literature yet.

## Country policies, economic and labour market outcomes during the first wave of the Covid-19 pandemic

The initial findings from the literature should be seen in the context of economic developments as well as policies introduced in response to the pandemic. From mid-March 2020 most of the European countries introduced strict policies, including school closures, workplace closures and travel bans. The introduced country policies varied, as did the impact of the crisis on national economies.

In the analyses, we capture Covid-19 policy responses by using the Stringency Index (country mean during the national fieldwork). It is a composite measure of nine metrics including: school closures; workplace closures; cancellation of public events; restrictions on public gatherings; closures of public transport; stay-at-home requirements; public information campaigns; restrictions on internal movements; and international travel controls, mean number of confirmed deaths, and duration of stay-at-home requirements (Hale et al. 2020). The Index is rescaled to a value from 0 to 100 (100 = strictest).

The Covid-19 Stringency Index in April reached above 90 (on the scale to 100) in Croatia, Cyprus, and Italy and above 80 in France, Malta, Romania, Slovenia, Greece, Poland, Spain, Portugal, Belgium, and Lithuania. It fell between June and August and increased again in October (Hale et al. 2020). Many countries also closed workplaces for all but key workers, particularly in the Western and Southern Europe (i.e. France, Italy, Spain, Portugal, Belgium, the Netherlands), while in Eastern Europe the workplace closures were required in some sectors (i.e. Germany, Denmark, Finland, Poland, Latvia). This was combined with the income support policies that covered more than 50% of the salary in case of most of the Western European countries (i.e. Sweden, Finland, Germany, France, Denmark, Spain, Latvia, Lithuania, and Estonia). In some countries the income support was below 50% of lost salary (i.e. Italy, Greece, Poland, Portugal).

Government policies triggered the responses of economies, which also differed between countries. The data for the first three quarters already indicate changes in the economy and on the labour market. The quarterly GDP growth, seasonally and calendar adjusted, shows that all countries faced GDP decline in the second quarter, and almost all in the third quarter of 2020. In the second quarter, countries which economies that suffered most were Italy, Denmark, France, Malta, and Portugal, that is countries that maintained stricter lockdown policies.

The economic situation also impacted the labour market. Employment rate, for workers aged 20–64 in the EU 27 countries, declined between Q3 and Q1 of 2020 by 0.2 p.p., while in the case of workers in age group 55–64, it increased slightly by 0.4 p.p. Labour market changes were also quite diverse between countries. As shown in Table 1, changes in the employment rate for the entire working age population were correlated with the situation of the population in age group 55–64. A decline of the employment rate of older workers was observed in Malta, Latvia, Luxembourg, and Finland, the employment rate of workers aged 55–64 years declined more than for the entire working age population, while also in Ireland, Lithuania, Spain, Italy, France, and the Netherlands there was a drop in employment for this age group. Interestingly, in Cyprus, Slovakia, Portugal, Slovenia, Czechia, Denmark, Belgium, and Estonia the employment rate of older workers increased, while it declined for the entire working age population.

**Table 1:** Country economic characteristics: change in the gross domestic product at market prices in the EU countries, Q2 2020, change in employment rate in the EU countries, Q2 2020 and HDI 2020. Source: Eurostat. Data extracted on 09/01/2021 15:31:01 from [ESTAT] (GDP, employment rate—for DE national data); HDI: (UNDP, 2020), Stringency Index: (Hale, Webster, Petherick, Phillips, & Kira, 2020)

	Employment rate 20–64 change Q2 2020	Employment rate 55–64 change Q2 2020	GDP change Q2 2020	HDI 2020	Stringency Index
Belgium	− 0.2	0.6	− 13.9	0.931	52.87
Bulgaria	1.6	2.1	− 8.6	0.816	37.85
Croatia	1.4	4.6	− 15.5	0.851	46.20
Cyprus	− 0.6	2.4	− 12.3	0.887	51.27
Czechia	− 0.4	1.2	− 10.8	0.900	37.09
Denmark	− 0.6	1.1	− 18.9	0.901	55.69
Estonia	− 1.7	0.1	− 5.4	0.892	32.13
Finland	0.2	− 0.2	− 6.2	0.938	36.08
France	− 0.5	− 0.4	− 18	0.892	52.04
Germany	− 1.4	− 0.6	− 11.2	0.947	58.80
Greece	− 0.5	2.5	− 8.0	0.940	49.27
Hungary	0.5	1.9	− 13.5	0.854	54.78
Italy	− 1.8	− 0.5	− 21.6	0.904	54.96
Latvia	− 0.2	− 0.8	− 8.6	0.866	49.47
Lithuania	− 2.8	− 0.7	− 4.6	0.882	29.70
Luxembourg	− 0.3	− 0.6	− 7.8	0.916	25.83
Malta	− 1.8	− 2.4	− 16.7	0.895	38.34
Poland	0.8	3.3	− 8	0.880	45.98
Portugal	− 1.1	1.5	− 16.4	0.864	69.90
Romania	0.6	2.0	− 10.3	0.828	43.93
Slovakia	− 0.6	2.1	− 8.3	0.860	38.69
Slovenia	− 0.9	1.3	− 12.9	0.917	43.21
Spain	− 0.6	− 0.5	− 9.2	0.944	56.46
Sweden	0.3	1.4	− 7.4	0.945	59.19
Switzerland	1.5	− 1.3	− 14.2	0.888	40.43

Seasonally and calendar adjusted data

Finally, the economic stress of the population can depend also on the overall level of country’s development before the Covid-19 outbreak. The Human Development Index (HDI) from 2020 (UNDP 2020) is an indicator that can be used to capture these differences. The HDI is a summary measure of average achievement in key dimensions of human development: a long and healthy life, being knowledgeable and have a decent standard of living. The HDI is the geometric mean of normalized indices for each of the three dimensions. At the dawn of the Covid-19 pandemic, the Human Development Index in the European countries participating in the SHARE Corona Telephone Survey ranged from 0.955 in the Netherlands to 0.816 in Bulgaria. It was highest in the countries in the Northern and South-Western Europe, while the lowest in Eastern Europe.

## Economic risks and financing consumption during the first wave of the pandemic—an analysis

### Data and methods

In our analysis, we examine the economic situation of people aged 50 or more in the European countries in the third quarter of 2020, based on the SHARE Corona Telephone Survey. Our sample covers 19,461 individuals from 25 countries, including EU Member States^1^ and Switzerland, who responded to questions related to their economic situation and related to receiving financial support during the prevalence of the stringency policies related to Covid-19 pandemic.

We analyse different symptoms of economic stress faced by the entire population of people aged 50 years or more or selected sub-populations, including:

For the entire population aged 50 or over:

- ability to make ends meet since the outbreak of the Covid-19 pandemic;
- receiving financial support since the outbreak of the Covid-19 pandemic.

For people who experienced difficulties with making ends meet (8621 respondents), we analyse the strategies of coping with current consumption that may have a longer-term impact on their financial situation in the future:

- postponing of the bills payment since the outbreak of the Covid-19 pandemic;
- dipping into savings to finance consumption of workers and pensioners since the outbreak of the Covid-19 pandemic;

Finally, for respondents who were employed before the Covid-19 outbreak (4666 respondents) we additionally look at the occurrence of job loss, since the outbreak of the Covid-19 pandemic, due to unemployment, lay off, or business closure.

In the analysis, we use the following individual socio-economic characteristics as explanatory variables: age group, sex, household size, educational attainment, labour market status as well as the economic situation of the household before the pandemic, measured by the ability of the household to make ends meet in 2017 (using the longitudinal information from Wave 7). The description of the sample used in the analysis is presented in Table 2. In the respective models sub-samples are used, depending on the analysed symptom of economic stress, which is explained above. As presented in Table 2a, the full sample includes more than 60% of women as respondents. Around half of respondents (more men than women) live in the two person households, while around a third (more in the case of women) live alone. The largest share of respondents has secondary education (ISCED 3–4). Majority of respondents are not working, above 40% are retired, slightly lower share is also not employed, which means that they may receive other types of social transfers (i.e. disability or survivor pensions) or they are inactive due to other reasons.

**Table 2:** Sample description. Source: Authors’ estimates using SHARE Corona Telephone Survey data Release 0.0.1 beta

a Full sample: financial support and making ends meet						
	Number of people			Structure		
	Men	Women	Total	Men	Women	Total
Total	6 776	12 621	19 397	34.93	65.07	100.00
				(% of respective total)		
*Household size*						
Single	1972	5035	7007	29.10	39.89	36.12
2 people	3545	5517	9062	52.32	43.71	46.72
3 or more people	1259	2069	3328	18.58	16.39	17.16
*Education*						
ISCED 0–2	1 760	4 032	5 792	25.97	31.95	29.86
ISCED 3–4	3 203	5 598	8 801	47.27	44.35	45.37
ISECD 5–6	1 813	2 991	4 804	26.76	23.70	24.77
*Economic status*						
Retired	3 131	5 649	8 780	46.21	44.76	45.26
Employed	1 016	1 666	2 682	14.99	13.20	13.83
Inactive	2 629	5 306	7 935	38.80	42.04	40.91
*Age group*						
50–54	52	206	258	0.77	1.63	1.33
55–59	778	1 460	2 238	11.48	11.57	11.54
60–64	1 392	2 496	3 888	20.54	19.78	20.04
65–69	1 603	2 858	4 461	23.66	22.64	23.00
70–74	1 601	2 854	4 455	23.63	22.61	22.97
75 and over	1 350	2 747	4 097	19.92	21.77	21.12
*Ability to make ends meet in 2017*						
Not difficult	4 197	6 643	10 840	61.94	52.63	55.88
Difficult	2 601	6 020	8 621	38.39	47.70	44.45
*Country*						
Germany	429	575	1 004	6.33	4.56	5.18
Sweden	231	288	519	3.41	2.28	2.68
Spain	189	382	571	2.79	3.03	2.94
Italy	417	810	1 227	6.15	6.42	6.33
France	274	511	785	4.04	4.05	4.05
Denmark	419	567	986	6.18	4.49	5.08
Greece	433	573	1 006	6.39	4.54	5.19
Switzerland	368	426	794	5.43	3.38	4.09
Belgium	721	1 003	1 724	10.64	7.95	8.89
Czechia	274	894	1 168	4.04	7.08	6.02
Poland	413	864	1 277	6.10	6.85	6.58
Luxembourg	169	200	369	2.49	1.58	1.90
Hungary	115	254	369	1.70	2.01	1.90
Portugal	121	252	373	1.79	2.00	1.92
Slovenia	331	788	1 119	4.88	6.24	5.77
Estonia	406	1 307	1 713	5.99	10.36	8.83
Croatia	291	511	802	4.29	4.05	4.13
Lithuania	125	481	606	1.84	3.81	3.12
Bulgaria	105	238	343	1.55	1.89	1.77
Cyprus	64	146	210	0.94	1.16	1.08
Finland	289	374	663	4.27	2.96	3.42
Latvia	119	362	481	1.76	2.87	2.48
Malta	93	189	282	1.37	1.50	1.45
Romania	218	424	642	3.22	3.36	3.31
Slovakia	184	244	428	2.72	1.93	2.21
*Receiving financial help*						
No	6 296	11 617	17 913	92.92	92.05	92.35
Yes	480	1 004	1 484	7.08	7.95	7.65
*Ability to make ends meet after pandemic*						
No	4 529	7 699	12 228	66.84	61.00	63.04
Yes	2 247	4 922	7 169	33.16	39.00	36.96
Postponing payment of the bills (people having difficulties to make ends meet)						
No	2 002	4 491	6 493	29.55	35.58	33.47
Yes	242	425	667	3.57	3.37	3.44
*Dipping into savings (people having difficulties to make ends meet)*						
No	1 805	4 010	5 815	26.64	31.77	29.98
Yes	439	909	1 348	6.48	7.20	6.95
Lost job—employed before the Covid-19 outbreak						
No	1 478	2 354	3 832	21.81	18.65	19.76
Yes	295	532	827	4.35	4.22	4.26

b Sub-sample: postponement of bills payment						
	Number of people			Structure		
	Men	Women	Total	Men	Women	Total
Total	2 244	4 916	7 160	31.34	68.66	100.00
				(% of respective total)		
*Household size*						
Single	729	2068	2797	32.49	42.07	39.06
2 people	995	1865	2860	44.34	37.94	39.94
3 or more people	520	983	1503	23.17	20.00	20.99
*Education*						
ISCED 0–2	783	2 104	2 887	34.89	42.80	40.32
ISCED 3–4	1 126	2 187	3 313	50.18	44.49	46.27
ISECD 5–6	335	625	960	14.93	12.71	13.41
*Economic status*						
Retired	920	2 032	2 952	41.00	41.33	41.23
Employed	263	466	729	11.72	9.48	10.18
Inactive	1 061	2 418	3 479	47.28	49.19	48.59
*Age group*						
50–54	28	98	126	1.25	1.99	1.76
55–59	299	592	891	13.32	12.04	12.44
60–64	497	980	1 477	22.15	19.93	20.63
65–69	553	1 064	1 617	24.64	21.64	22.58
70–74	470	1 121	1 591	20.94	22.80	22.22
75 and over	397	1 061	1 458	17.69	21.58	20.36
*Country*						
Germany	55	73	128	2.45	1.48	1.79
Sweden	22	42	64	0.98	0.85	0.89
Spain	40	97	137	2	2	2
Italy	174	415	589	7.75	8.44	8.23
France	47	114	161	2.09	2.32	2.25
Denmark	33	35	68	1.47	0.71	0.95
Greece	387	517	904	17.25	10.52	12.63
Switzerland	42	64	106	1.87	1.30	1.48
Belgium	103	216	319	4.59	4.39	4.46
Czechia	24	95	119	1.07	1.93	1.66
Poland	180	443	623	8.02	9.01	8.70
Luxembourg	14	20	34	0.62	0.41	0.47
Hungary	74	193	267	3.30	3.93	3.73
Portugal	58	138	196	2.58	2.81	2.74
Slovenia	137	396	533	6.11	8.06	7.44
Estonia	151	463	614	6.73	9.42	8.58
Croatia	173	326	499	7.71	6.63	6.97
Lithuania	48	215	263	2.14	4.37	3.67
Bulgaria	70	170	240	3.12	3.46	3.35
Cyprus	32	84	116	1.43	1.71	1.62
Finland	51	68	119	2.27	1.38	1.66
Latvia	57	202	259	2.54	4.11	3.62
Malta	36	75	111	1.60	1.53	1.55
Romania	146	310	456	6.51	6.31	6.37
Slovakia	90	145	235	4.01	2.95	3.28
*Receiving financial help*						
No	1987	4375	6362	88.55	89.00	88.85
Yes	257	541	798	11.45	11.00	11.15
*Dipping into savings (people having difficulties to make ends meet)*						
No	1 797	3 994	5 791	80.08	81.24	80.88
Yes	437	906	1 343	19.47	18.43	18.76
*Lost job—employed before the Covid-19 outbreak*						
No	337	578	915	15.02	11.76	12.78
Yes	130	249	379	5.79	5.07	5.29

c. Sub-sample: dipping into savings						
	Number of people			Structure		
	Men	Women	Total	Men	Women	Total
Total	2 237	4 903	7 140	31.33	68.67	100.00
				(% of respective total)		
*Household size*						
Single	726	2064	2790	32.45	42.10	39.08
2 people	992	1861	2853	44.35	37.96	39.96
3 or more people	519	978	1497	23.20	19.95	20.97
*Education*						
ISCED 0–2	783	2 098	2 881	35.00	42.79	40.35
ISCED 3–4	1 122	2 179	3 301	50.16	44.44	46.23
ISECD 5–6	332	626	958	14.84	12.77	13.42
*Economic status*						
Retired	917	2 026	2 943	40.99	41.32	41.22
Employed	259	465	724	11.58	9.48	10.14
Inactive	1 061	2 412	3 473	47.43	49.19	48.64
*Age group*						
50–54	28	98	126	1.25	2.00	1.76
55–59	295	592	887	13.19	12.07	12.42
60–64	497	976	1 473	22.22	19.91	20.63
65–69	553	1 062	1 615	24.72	21.66	22.62
70–74	469	1 118	1 587	20.97	22.80	22.23
75 and over	395	1 057	1 452	17.66	21.56	20.34
*Country*						
Germany	54	73	127	2.41	1.49	1.78
Sweden	22	42	64	0.98	0.86	0.90
Spain	41	97	138	1.83	1.98	1.93
Italy	173	414	587	7.73	8.44	8.22
France	46	112	158	2.06	2.28	2.21
Denmark	33	35	68	1.48	0.71	0.95
Greece	383	517	900	17.12	10.54	12.61
Switzerland	42	64	106	1.88	1.31	1.48
Belgium	103	216	319	4.60	4.41	4.47
Czechia	24	95	119	1.07	1.94	1.67
Poland	180	442	622	8.05	9.01	8.71
Luxembourg	14	20	34	0.63	0.41	0.48
Hungary	74	191	265	3.31	3.90	3.71
Portugal	58	138	196	2.59	2.81	2.75
Slovenia	137	397	534	6.12	8.10	7.48
Estonia	151	463	614	6.75	9.44	8.60
Croatia	173	325	498	7.73	6.63	6.97
Lithuania	47	211	258	2.10	4.30	3.61
Bulgaria	69	170	239	3.08	3.47	3.35
Cyprus	32	83	115	1.43	1.69	1.61
Finland	51	68	119	2.28	1.39	1.67
Latvia	58	200	258	2.59	4.08	3.61
Malta	36	75	111	1.61	1.53	1.55
Romania	146	310	456	6.53	6.32	6.39
Slovakia	90	145	235	4.02	2.96	3.29
*Receiving financial help*						
No	1 979	4 363	6 342	88.47	88.99	88.82
Yes	258	540	798	11.53	11.01	11.18
*Postponing payment of the bills (people having difficulties to make ends meet)*						
No	1 995	4 479	6 474	89.18	91.35	90.67
Yes	239	421	660	10.68	8.59	9.24
*Lost job—employed before the Covid-19 outbreak*						
No	336	577	913	15.02	11.77	12.79
Yes	125	250	375	5.59	5.10	5.25

d. Sub-sample: employed before Covid-19 outbreak						
	Number of people			Structure		
	Men	Women	Total	Men	Women	Total
Total	1 773	2 886	4 659	38.06	61.94	100.00
				(% of respective total)		
*Household size*						
Single	454	908	1362	25.61	31.46	29.23
2 people	831	1307	2138	46.87	45.29	45.89
3 or more people	488	671	1159	27.52	23.25	24.88
*Education*						
ISCED 0–2	454	908	1 362	25.61	31.46	29.23
ISCED 3–4	831	1 307	2 138	46.87	45.29	45.89
ISECD 5–6	488	671	1 159	27.52	23.25	24.88
*Economic status*						
Retired	195	265	460	11.00	9.18	9.87
Employed	874	1 499	2 373	49.29	51.94	50.93
Inactive	704	1 122	1 826	39.71	38.88	39.19
*Age group*						
50–54	39	142	181	2.20	4.92	3.88
55–59	548	1 004	1 552	30.91	34.79	33.31
60–64	688	1 115	1 803	38.80	38.63	38.70
65–69	300	415	715	16.92	14.38	15.35
70–74	137	148	285	7.73	5.13	6.12
75 and over	61	62	123	3.44	2.15	2.64
*Ability to make ends meet in 2017*						
Not difficult	1 306	2 058	3 364	73.66	71.31	72.20
Difficult	467	828	1 295	26.34	28.69	27.80
*Country*						
Germany	129	168	297	7.28	5.82	6.37
Sweden	70	60	130	3.95	2.08	2.79
Spain	20	36	56	1.13	1.25	1.20
Italy	102	133	235	5.75	4.61	5.04
France	40	89	129	2.26	3.08	2.77
Denmark	192	192	384	10.83	6.65	8.24
Greece	81	62	143	4.57	2.15	3.07
Switzerland	114	124	238	6.43	4.30	5.11
Belgium	186	253	439	10.49	8.77	9.42
Czechia	61	163	224	3.44	5.65	4.81
Poland	138	209	347	7.78	7.24	7.45
Luxembourg	29	21	50	1.64	0.73	1.07
Hungary	15	29	44	0.85	1.00	0.94
Portugal	17	47	64	0.96	1.63	1.37
Slovenia	37	93	130	2.09	3.22	2.79
Estonia	139	472	611	7.84	16.35	13.11
Croatia	39	80	119	2.20	2.77	2.55
Lithuania	44	171	215	2.48	5.93	4.61
Bulgaria	42	56	98	2.37	1.94	2.10
Cyprus	15	30	45	0.85	1.04	0.97
Finland	99	141	240	5.58	4.89	5.15
Latvia	46	104	150	2.59	3.60	3.22
Malta	24	24	48	1.35	0.83	1.03
Romania	37	49	86	2.09	1.70	1.85
Slovakia	57	80	137	3.21	2.77	2.94
*Receiving financial help*						
No	1 555	2 517	4 072	87.70	87.21	87.40
Yes	218	369	587	12.30	12.79	12.60
*Ability to make ends meet after pandemic*						
No	1 306	2 058	3 364	73.66	71.31	72.20
Yes	467	828	1 295	26.34	28.69	27.80
*Postponing payment of the bills (people having difficulties to make ends meet)*						
No	381	720	1 101	21.49	24.95	23.63
Yes	86	107	193	4.85	3.71	4.14
*Dipping into savings (people having difficulties to make ends meet)*						
No	381	720	1 101	21.49	24.95	23.63
Yes	86	107	193	4.85	3.71	4.14

The sub-sample for postponed bills payment (Table 2b) comprises 68.7% of women (more than in the full sample); around 47% lives in two persons households. Around 46% of people have secondary education (which is similar to the full sample). More respondents than in the case of the full sample are inactive, but not retired (almost 49%), the age structure is also similar to the full sample. More people than in the full sample (11%) declared that they received financial help. In this sub-sample almost a fifth of respondents who postponed payment of bills also dipped into their savings. The second sub-sample includes people who dipped into their savings among whom, more than in the total sample lived in single households (almost 40%) and less in the two person households. Structure of educational attainment and the age structure are the same as in the full sample. Again, in this sub-sample more people are inactive but not retired (almost 49%). Around 11% received financial help and almost more than 9% dipped into their savings (which is more than in the full sample). Finally, in the sub-sample of people who were employed before the Covid-19 outbreak there are less women (61%). Household size and education structure are like in the full sample. Compared to the full sample there are more people who are employed and less people who are retired. This sub-sample also comprises a younger population compared to the full sample. Less people had difficulties in making ends meet, but more dipped into their savings.

Given the differences observed between the countries, we use the following country characteristics as explanatory variables, to verify how they influence the economic stress faced by people aged 50 or over:

- the overall level of development before the Covid-19 outbreak, measured by the Human Development Index (HDI) from 2020 (UNDP 2020);
- the short-term economic response to the crisis, measured the changes in the quarterly GDP and employment rate (seasonally adjusted) between first and second quarter of 2020;
- Covid-19 policy responses measured by the Stringency Index (country mean during the national fieldwork) (Hale et al. 2020).

The correlations between considered characteristics at country level are not high (Table 3). Only in the case of GDP change in the Q2 of 2020 and the Stringency Index the correlation coefficient is relatively high (− 0.58). The collinearity test between variables indicates that they can be used in the regression model.

**Table 3:** Country characteristics: correlation matrix. Source: Authors’ estimates

	Employment rate change Q2 2021	GDP change Q2 2020	HDI 2020	Stringency Index
Employment rate change Q2 2021	1.000			
GDP change Q2 2020	− 0.007	1.000		
HDI 2020	− 0.325	0.032	1.000	
Stringency Index	0.063	− 0.578	0.268	1.000

### *How the economic situation of people 50* + *differed between countries during the first wave of the Covid-19 pandemic?*

To assess the risk of the analysed symptoms of economic stress we estimated logistic regression models with two groups of variables, according to the following equation:

$$\ln \left[ {\frac{\pi }{1 - \pi }} \right] = \alpha + \sum\limits_{{\text{j}}} {\beta_{{\text{j}}} X_{{{\text{ij}}}} } + \sum\limits_{{\text{k}}} {\beta_{{\text{k}}} X_{{{\text{i}}\left( {{\text{gender}}} \right)}} X_{{{\text{i}}\left( {{\text{age}}\;{\text{group}}} \right)}} } + \sum\limits_{{\text{l}}} {\gamma_{{\text{l}}} Y_{{{\text{cl}}}} }$$

where $\beta_{{\text{j}}}$ are coefficients related to individual characteristics, $\beta_{{\text{k}}}$ are coefficients related to interactions between gender and age groups, and $\gamma_{{\text{l}}}$ are coefficients related to country characteristics.

The obtained results reveal significant differences in the economic situation of older people since the outbreak of the Covid-19 pandemic, in case of all five analysed symptoms of economic stress including: receiving financial support (both in the form of public transfer or help from family and friends) and increased difficulties to make ends meet for the entire population. In the case of the economically vulnerable group (that is people who face difficulties in making ends meet) the risk of postponing of the payment of bills and using savings to finance current expenditure varies between countries. Finally, the risk of job loss among those who were employed prior to the pandemic also depends on the country of residence characteristics (Table 4).

**Table 4:** Symptoms of economic stress—country means. Source: Authors’ estimates using SHARE Corona Telephone Survey data Release 0.0.1 beta

Country	Ends meet	Financial support	Bills	Savings	Lost job
Belgium	0.200	0.110	0.175	0.349	0.251
Bulgaria	0.703	0.056	0.132	0.279	0.202
Croatia	0.624	0.042	0.072	0.125	0.092
Cyprus	0.578	0.111	0.179	0.259	0.377
Czechia	0.103	0.093	0.046	0.529	0.030
Denmark	0.079	0.025	0.051	0.415	0.080
Estonia	0.384	0.065	0.038	0.120	0.088
Finland	0.154	0.040	0.178	0.275	0.157
France	0.233	0.128	0.159	0.330	0.388
Germany	0.177	0.046	0.069	0.284	0.159
Greece	0.909	0.110	0.247	0.186	0.377
Hungary	0.792	0.042	0.099	0.207	0.139
Italy	0.524	0.143	0.198	0.393	0.319
Latvia	0.552	0.048	0.165	0.097	0.074
Lithuania	0.423	0.047	0.036	0.160	0.186
Luxembourg	0.107	0.039	0.101	0.256	0.290
Malta	0.370	0.058	0.094	0.212	0.172
Poland	0.502	0.067	0.078	0.211	0.059
Portugal	0.626	0.047	0.052	0.135	0.240
Romania	0.742	0.049	0.078	0.118	0.124
Slovakia	0.586	0.041	0.044	0.151	0.217
Slovenia	0.483	0.570	0.033	0.081	0.331
Spain	0.282	0.106	0.070	0.314	0.172
Sweden	0.119	0.028	0.084	0.377	0.116
Switzerland	0.152	0.058	0.069	0.401	0.268

**Fig. 1:**
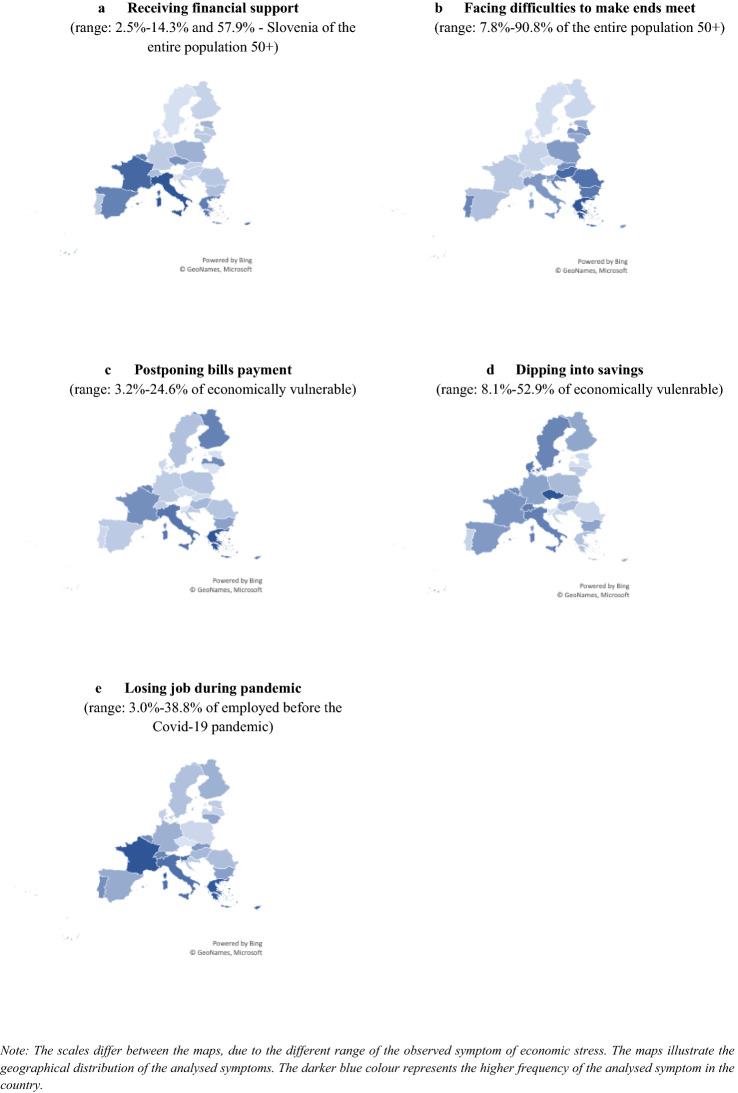
Analysed symptoms of economic stress by countries (means). Source: Authors’ estimates using SHARE Corona Telephone Survey data Release 0.0.1 beta

For illustration of the distribution of the analysed symptoms of economic stress, we also present them on maps (Fig. 1).

During the first wave of the pandemic, relatively few respondents in general reported receiving some kind of financial support (around 8%). In this case, there is one outlier–Slovenia, where almost 57% of respondents received financial support. In the rest of the countries such help was received by more than 10% of respondents in Italy, France, Cyprus, Belgium, and Greece, while in Denmark, Sweden, Luxembourg, and Finland (that is mainly Northern countries) only less than 5% of respondents declared receiving financial support (Table 4, Fig. 1a).

More than a third of the surveyed population declares that their ability to make ends meet worsened after the outbreak of the pandemic. Again, people living in the Southern Europe as well as most of the “new” EU countries, faced such difficulties more often, with more than 90% of people indicating worsening economic situation in Greece and more than 70% in Hungary, Romania, and Bulgaria. In Denmark, Czechia, Luxembourg, and Sweden the economic situation of the households of people 50 and over was better; less than 15% of respondents have reported problems with making ends meet (Table 4, Fig. 1b).

Among those who face economic difficulties, arrears in the payment of bills are also an indicator of economic difficulties, but it also signals the household strategies to deal with the financing of the current consumption. Around 12% of respondents that face economic difficulties indicated that they postponed the regular payment of bills. SHARE results again show large differences between countries, which are like the ones observed in case of job loss. The largest share of people postponing bills payment was noted in Greece, Italy, Cyprus, Finland, Belgium, and Latvia, while in Slovenia, Lithuania, Estonia, Slovakia, and Czechia (Table 4, Fig. 1c). Another strategy to deal with income loss during the crisis is to use savings to finance current consumption–around 27% of respondents in the economically vulnerable group admitted that they dipped into their savings. Also, in this case we observe large differences between countries. Namely, more than 40% of the economically vulnerable group in Switzerland, Denmark, and Czechia dipped into their savings, while in Slovenia, Latvia, Romania, and Estonia it was around 10% or less (Table 4, Fig. 1d). Such strategy seems to be popular particularly in case of countries in which people have larger savings, while fewer respondents from the “new” EU countries (with exception of Czechia) reported such source of financing of their current expenses. However, as shown by the Eurofound research, in many of these countries, households do not have savings that could be used in the time of crisis, which can be a potential explanation of the observed differences.

Finally, when we focus on the group that was economically active before the outbreak of the pandemic, one in five workers aged 50 and over in the analysed countries lost their job after the outbreak of the pandemic. The incidence of job loss was lowest in Czechia, Poland, Latvia, and Denmark and the highest in France, Greece, Slovenia, Cyprus, and Italy. Overall, the risk of job loss was higher in the Southern European countries, but also many conservative ones (France, Luxembourg, Switzerland, Belgium). The risk of job loss was lower in Northern Europe, but also in many Central and Eastern European countries that joined the EU in 2004 and later (Fig. 1e). Results from the SHARE survey are broadly consistent with the LFS results discussed earlier. Interestingly, the risk of job loss in some of the “new” member states is lower compared to the earlier crises (i.e. Baltic states during the Great Recession or Poland at the turn of the century). One of the possible explanations is that the labour market adjustments in the past make employers more aware of the long-term challenges related to recruitment of workers, particularly in the countries where working age population in ageing.

Comparison of the economic difficulties experienced by older people since the outbreak of the Covid-19 pandemic by countries reveals some similarities and differences. Older people living in Southern European countries, both in the “old” and “new” European countries, face the largest difficulties in making ends meet during the pandemic. At the same time, despite a steep drop in the GDP, respondents in France and Denmark face less difficulties to make ends meet. Yet, the risk of job loss in France was the highest, but to some extent it was offset by the provision of financial support. In general, we also observe that people living in the “old” European Member States faced higher risk of job loss and used their savings more often, compared to the “new” countries. In those countries, where the incidence of job loss was higher, people also postponed payment of bills and more frequently received financial support. It is also worth to notice that despite a smaller exposure to economic risks, in some countries, people declared using their savings more frequently (i.e. Sweden and Italy). Finally, while people in the “new” Member States seem to have a lower risk of job loss, they more frequently indicated increased difficulties in making ends meet, which shows that in general they are more exposed to the economic hardships caused by unexpected shocks. We also observe that the share of people receiving financial support was lower in the “new” countries, with notable exception of Slovenia, which can be also linked to a lower share of people that lost their jobs during the first months of the pandemic.

The tetrachoric correlations (r_tet_)^2^ between the analysed symptoms of economic stress presented in Table 5 show that there is a positive correlation between these symptoms. However, these relationships are not very strong. The r_tet_ coefficients are the largest between job loss and receiving financial support (0.52), which is intuitive, as well as between job loss and postponing of the bills payment (0.48), which may indicate that in case of job loss people first tend to finance their current consumption needs at the expense of rising indebtedness. The r_tet_ coefficient between postponing bills payment and dipping into savings (0.43) indicates that those that are most economically vulnerable tend to combine these two strategies to finance the current consumption. The r_tet_ coefficients between discussed variables justify the modelling of all five symptoms of economic stress separately.

**Table 5:** Symptoms of economic stress of the population 50 + in Europe: tetrachoric correlation matrix. Source: Authors’ estimates using SHARE Corona Telephone Survey data Release 0.0.1 beta

		Making ends meet	Financial support	Postponing bills	Dipping into savings	Lost job
Making ends meet	rho	1.00				
	n	19 397				
	2-sided exact P	0.00				
Financial support	rho	0.228	1.00			
	n	19 397	19 397			
	2-sided exact P	0.00				
Postponing bills	rho		0.145	1.000		
	n		7 160	7 160		
	2-sided exact P		0.00			
Dipping into savings	rho		0.118	0.424	1.00	
	n		7 140	7 134	7 140	
	2-sided exact P		0.00	0.00		
Lost job	rho	0.336	0.520	0.401	0.479	1.00
	n	4 659	4 659	1 294	1 288	4 659
	2-sided exact P	0.00	0.00	0.00	0.00	

### Individual and country characteristics—what explains the economic difficulties experienced since the outbreak of the Covid-19 pandemic?

#### Facing financial difficulties

To verify how the observed symptoms of economic risks depend on individual characteristics as well as differences in country economic situation and implemented policies, we performed logistic regression models for each of the five analysed symptoms of economic stress as dependent variables. The explanatory variables include individual socio-economic and country characteristics, as presented in Sect. 2. We also consider interactions between age and sex. The results of the logistic regression models are shown in Table 6 (regression coefficients) and Table 7 (average marginal effects), which indicate how the probability of exposure to the specific economic risk changes depending on individual and country characteristics.

**Table 6:** Logistic regression coefficients: symptoms of economic stress. Source: Authors’ estimates using SHARE Corona Telephone Survey data Release 0.0.1 beta

	(1)	(2)	(3)	(4)	(5)
	Financial support	Difficulties to make ends meet	Postponing bills payment	Dipping into savings	Lost job
*Sex (baseline: male)*					
Female	− 0.490*	− 0.139	− 0.398	− 0.00833	0.506
	(0.282)	(0.126)	(0.288)	(0.233)	(0.357)
Age group (baseline 65–69) 50–54	0.507	0.169	− 1.261	0.929	− 0.564
	(0.679)	(0.503)	(0.853)	(0.583)	(0.784)
55–59	0.969***	0.548***	0.175	0.691**	− 0.215
	(0.349)	(0.194)	(0.347)	(0.304)	(0.360)
60–64	0.315	− 0.0815	− 0.0757	0.119	− 0.0708
	(0.398)	(0.200)	(0.347)	(0.359)	(0.330)
70–74	− 0.694**	− 0.243	− 0.743**	− 0.610**	− 1.067*
	(0.341)	(0.154)	(0.365)	(0.284)	(0.548)
75 or over	− 0.762*	− 0.339**	− 0.619*	− 0.786***	0.402
	(0.429)	(0.157)	(0.338)	(0.301)	(0.682)
Sex and age interactions Women # 50–54	0.498	1.052*	1.278	− 1.372*	− 0.341
	(0.780)	(0.618)	(0.989)	(0.746)	(0.924)
Women # 55–59	0.219	0.0782	0.771*	− 0.0334	− 0.440
	(0.387)	(0.238)	(0.450)	(0.376)	(0.463)
Women # 60–64	0.782*	0.549**	0.333	0.213	− 0.198
	(0.424)	(0.251)	(0.426)	(0.408)	(0.429)
Women # 70–74	0.482	0.384**	0.493	0.540	1.854***
	(0.437)	(0.186)	(0.443)	(0.344)	(0.702)
Women # 75 or over	0.201	0.0342	0.175	0.701**	0.671
	(0.492)	(0.189)	(0.443)	(0.355)	(0.909)
Household size (baseline: 2 people) 1 person	− 0.275	0.490***	− 0.180	0.0207	0.163
	(0.200)	(0.0911)	(0.223)	(0.155)	(0.206)
3 or more people	0.162	0.291**	0.291	0.352*	− 0.362
	(0.219)	(0.125)	(0.240)	(0.191)	(0.228)
Educational attainment (baseline: ISCED 3–4—secondary)	0.223	0.465***	0.161	− 0.159	0.104
ISCED 0–2 below secondary	(0.216)	(0.104)	(0.237)	(0.165)	(0.247)
ISCED 5–6 higher	− 0.0634	− 0.591***	0.386	0.180	− 0.796***
	(0.210)	(0.111)	(0.297)	(0.221)	(0.190)
Economic status (baseline: retired) Employed	0.904**	− 0.433***	0.848***	0.366	0.0111
	(0.362)	(0.153)	(0.294)	(0.286)	(0.262)
Not employed or retired	0.435	0.201**	0.467**	0.110	0.179
	(0.344)	(0.0881)	(0.182)	(0.213)	(0.264)
Difficulty to make ends meet in 2017 (SHARE Wave 7)	0.306*	1.925***	0.654**	− 0.192	0.502***
	(0.182)	(0.0862)	(0.267)	(0.160)	(0.188)
Employment rate 20–64 change in Q2 2020	0.0950	− 0.177***	0.164*	0.179**	0.0940
	(0.0815)	(0.0421)	(0.0972)	(0.0810)	(0.0924)
GDP change in Q2 2020	− 0.0826***	0.0398***	− 0.0746***	− 0.0802***	− 0.112***
	(0.0175)	(0.0100)	(0.0229)	(0.0188)	(0.0194)
Human Development Index 2020	7.536**	− 17.90***	7.150**	10.51***	3.503
	(3.132)	(1.637)	(3.192)	(3.187)	(3.212)
Stringency Index (country mean during national fieldwork)	− 0.0354***	0.0150**	− 0.0345**	− 0.0188	− 0.0102
	(0.0115)	(0.00687)	(0.0163)	(0.0153)	(0.0121)
Observations included in the model	19,380	19,380	7,154	7,134	4,649
Pseudo-R-squared (model with both individual and country char.)	0.1155	0.2632	0.0907	0.0729	0.0842
Pseudo-R-squared (model with individual characteristics only)	0.1021	0.2353	0.0798	0.0529	0.0503
Pseudo-R-squared (model with country characteristics only)	0.0212	0.0750	0.0243	0.0314	0.0380

^***^p < 0.01, ** *p* <  0.05, *p < 0.1

**Table 7:** Logistic regression average marginal effects: symptoms of economic stress. Source: Authors’ estimates using SHARE Corona Telephone Survey data Release 0.0.1 beta

	(1)	(2)	(3)	(4)	(5)
	Financial support	Difficulties to make ends meet	Postponing bills payment	Dipping into savings	Lost job
female	− 0.005**	0.018**	0.011**	0.019**	0.045**
*Age group (baseline 65–69)*					
50–54	0.048**	0.127**	− 0.031**	0.029*	− 0.153**
55–59	0.082**	0.097**	0.073**	0.129**	− 0.072**
60–64	0.048**	0.039**	0.011**	0.050**	− 0.053**
70–74	− 0.017***	− 0.001**	− 0.033**	− 0.042**	0.005*
75 or over	− 0.023***	− 0.044**	− 0.039**	− 0.052**	0.157*
Household size (baseline: 2 people)*					
1 person	− 0.018**	0.074**	− 0.017**	0.003**	0.022**
3 or more people	0.013**	0.044**	0.029**	0.061**	− 0.050**
Educational attainment (baseline: ISCED 3–4)					
ISCED 0–2	0.017**	0.076**	0.015**	− 0.032**	0.027**
ISCED 5–6	− 0.003**	− 0.091**	0.040**	0.032**	− 0.099**
Economic status (baseline: retired)					
Employed	0.063**	− 0.066**	0.083**	0.069*	0.019**
Not employed or retired	0.026**	0.030**	0.038**	0.018**	0.015**
Difficulty to make ends meet in 2017 (SHARE Wave 7)	0.022**	0.370***	0.057**	− 0.036***	0.064**
Employment rate 20–64 change in Q2 2020	0.008***	− 0.028***	0.016***	0.033**	0.021**
GDP change in Q2 2020	− 0.006***	0.006***	− 0.007***	− 0.014***	− 0.018***
Human Development Index 2020	0.570	− 2.824	0.696	1.878	0.793
Stringency Index (country mean during national fieldwork)	− 0.003***	0.002***	− 0.003***	− 0.003***	− 0.002***

^***^p < 0.01, ** *p* <  0.05, *p < 0.1

As shown in Table 6, the samples included in the models depend on the explained variable. In the case of the first two models explaining the receiving of financial support and the difficulty in making ends meet, the entire sample is used in the model. For models explaining the postponement bills payment and dipping into savings, the sample is restricted to people who had difficulties in making ends meet and responded to the questions related to explanatory variables (7154 and 7134 people, respectively). In the case of the model explaining the job loss, we include only people who worked before the outbreak of the Covid-19 pandemic (4,649 people).

The models confirm that socio-economic characteristics of individuals as well as the country characteristics are statistically significant when explaining various symptoms of economic stress.

Let us first look which individual socio-economic characteristics are significant when explaining the economic stress faced by SHARE Corona Telephone Survey respondents during the first wave of the Covid-19 pandemic (as shown in Table 6) in the first four models, related to symptoms of economic stress such as receiving financial support, facing difficulties to make ends meet and (within the latter group) those postponing payment of the bills and dipping into savings.

An important distinction affecting economic risk faced by the SHARE Corona Telephone Survey respondents is age and to a lesser extent sex. Compared to those in age group 65–69 years (baseline), people in age group 55–59 received financial support more frequently (the average marginal effect in Table 7 shows that it was by 8.2% more), while those in age groups 70–74 years and 75 year or over less frequently (average marginal effects equal to − 1,7% and − 2.3%, respectively). Similar results are observed in case of making ends meet. Among those most vulnerable, those in age group 55–59 were more likely to dip into their savings, while people above 70 years of age less frequently postponed the bills payment and dipped into savings.

People living alone face higher difficulties in making ends meet compared to couples (with average marginal effect of 7.4%). At the same time, those living in larger households also have a higher risk of such difficulties (by 4.4%). Those living in larger households (3 or more people) and struggling to make ends meet were also more likely to dip into their savings (by 6.1%).

The experienced symptoms of economic stress also depend on the educational attainment. Compared to those with secondary education (ISCED 3–4), people with lower educational attainment have 7.6% more chances of facing difficulties in making ends meet, while those with higher education have 9.1% less chances of such situation.

Compared to the retired people, those still employed were more likely to receive financial support (by 6.3%); they also had smaller difficulties in making ends meet (by 6.6%). Among those more vulnerable, employees were more likely to postpone the bills payment (by 8.3%). Those who were neither working nor retired also more frequently postponed the bills payment (by 3.8%).

Finally, earlier exposure to economic difficulties understood as the difficulty in making ends meet observed in 2017 also increased chances to face economic risks during the Covid-19 pandemic. They have much bigger difficulties in making ends meet after the pandemic outbreak (by 37.0%), as well as higher chances to receive financial support (by 6.2%). Those more vulnerable earlier also more frequently postponed their bills payment (by 5.6%).

Our results also reveal the interactions between age group and sex, which are shown in regression results in Table 6. Average marginal effects for these interactions are presented in Table 8. While women in general were only less likely to receive support, compared to men, the age and sex interactions reveal that in some age groups women were more vulnerable. Women in age group 60–64 were more likely to receive financial support (by 2.5%), but also struggled more to make ends meet (by 6.5%), compared to men in these age groups. Among the more vulnerable, women in age group 55–59 more frequently postponed bills payment (by 5%). Finally, women aged 75 or over more frequently dipped into their savings if they had difficulties to make ends meet (by 9.1%).

**Table 8:** Logistic regression average marginal effects: symptoms of economic stress for age group and sex interactions. Source: Authors’ estimates using SHARE Corona Telephone Survey data Release 0.0.1 beta

	(1)	(2)	(3)	(4)	(5)
	Financial support	Difficulties to make ends meet	Postponing bills payment	Dipping into savings	Lost job
male (base outcome)					
female at					
55–54	0.001	0.152	0.050	− 0.256	0.020
55–59	− 0.030	− 0.010	0.050	− 0.009	0.009
60–64	0.025	0.065	− 0.006	0.038	0.050
65–69	− 0.023	− 0.021	− 0.036	− 0.001	0.089
70–74	0.000	0.037	0.006	0.074	0.381
75 and over	− 0.008	− 0.015	− 0.013	0.091	0.249

dy/dx for factor levels is the discrete change from the base level

Results of the models also confirm that the country characteristics are related to the observed symptoms of economic stress. The drop in the GDP observed in the Q2 of 2020 led to the increased share of people receiving financial support (the average marginal effect of 0.6% per 1 pp of the GDP decline, as presented in Table 7), slightly lower risk of facing difficulties to make ends meet (by 0.6%) and among those most vulnerable also higher share of those postponing the bills payment (by 0.7%) and dipping into savings (by 1.4%).

Additionally, the worsening labour market situation measured by the employment rate decline, was also associated with higher share of people postponing the bills payment and dipping into savings, among those most vulnerable. Overall country’s level of development also affects the risk of facing economic difficulties. Higher HDI increased chances to receive financial support (by 5.7% per 0.1 point of HDI), but also to a large extent reduced the risk of difficulties in making ends meet (by 28%). This means that countries with a higher level of social and economic growth are more resilient to economic stress and at the same time have a potential to provide financial support to a larger share of the population. At the same time, those most vulnerable were more likely to dip into savings or postpone bills payment in countries with a higher HDI. We can hypothesise that they treat their economic difficulties as a transitory situation and believe that with the return to the growth path their situation will improve again.

We observe a direct impact of introduced policies captured by the Stringency Index. Higher government policy stringency is associated with lower chances of receiving financial support, while higher ones in facing difficulties to make ends meet. At the same time, those with economic difficulties less frequently decided to postpone their bills payments in countries that introduced more strict stringency policies.

#### Losing a job

As the sub-population of people at risk of job loss differs from the entire analysed population, we discuss the results of the logistic regressions for this symptom separately. Results of the logistic regression for the job loss are also included in Tables 6, 7, and 8.

The risk of job loss was less frequent among those in age group 70–74 (compared to those in age group 65–69); women in this age group were more likely to lose a job compared to men, which is shown in interaction effects. Job loss was also less frequent among people with higher educational attainment. People with this level of education had 9.9% less probability of losing a job, compared to those with secondary education. The job loss among those who were employed prior to the Covid-19 outbreak and already faced economic risk was higher by 6.4% compared to those that did not have such difficulties earlier.

The immediate response of the economy, measured by the GDP decline, was also associated with a higher risk of job loss (by 1.3%). Overall change in employment rate (20–64 years) affected the risk of job loss among those 50 and over (by 4%). Interestingly, the share of those that lost a job was higher in countries with higher HDI. The short-term labour market adjustment was therefore combined with the higher ability of these countries to provide financial support and also higher resilience of the population to difficulties with making ends meet.

To assess the explanatory power of individual and country characteristics, separate models with only individual and only country characteristics were estimated. The values of pseudo-R-squared for the models are presented at the end of Table 6. Overall, individual characteristics explain more observed variance than country ones, particularly in the case of models explaining the chances to receive financial support or difficulties to make ends meet. Yet, the combination of both individual and country characteristics improves the model fit.

## Discussion and conclusions

The results of the SHARE Corona Telephone Survey reveal differences in the economic risks faced by individuals both with respect to their individual characteristics, but also countries which they live in. This indicates that immediate country policies, their impact on the economic growth and labour market, but also country development affect economic situation of the older population in Europe.

Stringent lockdown policies, including workplace closures, led to increased risk of making ends meet, but also lower chances of receiving financial support. While in general the governments introduced policies to replace the lost income of people affected by workplace closures, it seems that in the case of more stringent policies, the financial support was less available.

The income support was mainly addressed to the working population. Others (including pensioners) received their social insurance transfers, and therefore, their incomes did not suffer as much. Increased vulnerability among people aged 50 and over, who are not receiving pensions, triggered adjustments such as postponing the bills payments or dipping into savings. We might hypothesise that those that are still economically active treat the income loss as temporary, and they assume that they will regain their income, so they will be able to make up for the arrears against their future income. If such strategies are continued, the economic situation of the older population might worsen. Some of the decisions, such as using savings to finance current consumption, particularly for those who will soon reach retirement age, might also have long-term consequences on their future income after retirement, as they will not be able to rebuild their savings before retirement.

The SHARE Corona Telephone Survey results also show that the economic vulnerability of people living in countries with lower Human Development Index is higher. This can relate to the relatively weaker welfare state provisions (observed for example by smaller income support provided during the lockdown). At the same time, people living in these countries might not have sufficient savings to use for their consumption in the case of reduced household income.

Contrary to our expectations, we see that the employed people in general assessed their economic situation slightly better than pensioners, but they more frequently resorted to strategies such as postponement of the payment of the bills as well as they received financial support to cover their wages, which could contribute to the relatively weaker influence on the ability to make end meet.

However, it should be noted that these findings capture only the first reaction to the policy measures introduced at the outbreak of the Covid-19 pandemic and there is a need to monitor the long-term effects of the crisis on the labour market and employment, as longer periods of lockdown and workplace closures, as well as worsening public finance situation can further affect the economic risks faced by the older workers and pensioners.

## Footnotes

1 The sample does not include Austria, Ireland, and the Netherlands. The first two countries did not participate in the SHARE Corona Telephone Survey, and in the case of the Netherlands, there is no information on the economic status of respondents in 2017, which is one of the variables used in the analysis.

2 We use tetrachoric correlations due to the dichotomic type of variables.
